# Lack of Neuroprotection with a Single Intravenous Infusion of Human Amnion Epithelial Cells after Severe Hypoxia–Ischemia in Near-Term Fetal Sheep

**DOI:** 10.3390/ijms23158393

**Published:** 2022-07-29

**Authors:** Joanne O. Davidson, Lotte G. van den Heuij, Simerdeep K. Dhillon, Suzanne L. Miller, Rebecca Lim, Graham Jenkin, Alistair J. Gunn, Laura Bennet

**Affiliations:** 1 Fetal Physiology and Neuroscience Group, Department of Physiology, The University of Auckland, Auckland 1010, New Zealand; heuijl@gmail.com (L.G.v.d.H.); s.dhillon@auckland.ac.nz (S.K.D.); aj.gunn@auckland.ac.nz (A.J.G.); l.bennet@auckland.ac.nz (L.B.); 2 The Ritchie Centre, Hudson Institute of Medical Research, Clayton 3168, Australia; suzie.miller@monash.edu (S.L.M.); rebecca.lim@monash.edu (R.L.); graham.jenkin@monash.edu (G.J.); 3 Department of Obstetrics and Gynaecology, Monash University, Clayton 3800, Australia

**Keywords:** human amnion epithelial cells, hypoxia ischemia, neuroprotection, fetal sheep, inflammation

## Abstract

Background: Hypoxic–ischemic encephalopathy (HIE) around the time of birth results from loss of oxygen (hypoxia) and blood supply (ischemia). Exogenous infusion of multi-potential cells, including human amnion epithelial cells (hAECs), can reduce hypoxic–ischemic (HI) brain injury. However, there are few data on treatment of severe HI in large animal paradigms at term. The aim of the current study was to determine whether infusion of hAECs early after injury may reduce brain damage after ischemia in near-term fetal sheep. Methods: Chronically instrumented fetal sheep (0.85 gestation) received 30 min of global cerebral ischemia followed by intravenous infusion of hAECs from 2 h after the end of ischemia (ischemia-hAEC, n = 6) or saline (ischemia-vehicle, n = 7). Sham control animals received sham ischemia with vehicle infusion (sham control, n = 8). Results: Ischemia was associated with significant suppression of EEG power and spectral edge frequency until the end of the experiment and a secondary rise in cortical impedance from 24 to 72 h, which were not attenuated by hAEC administration. Ischemia was associated with loss of neurons in the cortex, thalamus, striatum and hippocampus, loss of white matter oligodendrocytes and increased microglial numbers in the white matter, which were not affected by hAEC infusion. Conclusions: A single intravenous administration of hAECs did not reduce electrographic or histological brain damage after 30 min of global cerebral ischemia in near-term fetal sheep.

## 1. Introduction

Hypoxia–ischemia (HI) before, during or around the time of birth is the primary cause of hypoxic–ischemic encephalopathy (HIE), which occurs in approximately 2/1000 live births in high resource settings and approximately 26/1000 live births in low resource settings [1], and is associated with a high rate of mortality and morbidity in survivors, including lifelong physical and mental disabilities [2,3]. Therapeutic hypothermia (TH) is now established as a neuroprotective treatment for term infants with moderate to severe HIE in high resource settings [4,5]. However, protection is only partial, and many infants still survive with brain damage and disability despite treatment [4,5]. Furthermore, the use of TH in low and middle income countries remains controversial, given that the recent HELIX trial showed that TH was not associated with any effect on combined death and disability and was associated with a significant increase in the risk of death in infants with moderate to severe HIE in these settings [6]. Therefore, new stand-alone and/or additive therapies are required to reduce the burden of injury associated with HIE throughout the world [4,5].

Stem cells have the potential to be neuroprotective through a variety of mechanisms including immunomodulation, which may reduce injurious inflammation, and release of factors such as neurotrophins, which are neuroprotective and can promote proliferation, maturation, and differentiation of new cells [7,8,9,10]. Stem cells may also be able to extend the window of opportunity from the current 6 h for TH implementation, to anywhere from 24 h to 14 days after the initial insult [11].

Human amniotic epithelial cells (hAECs) have many benefits over other types of cells as a potential therapy, including being widely available, ethically acceptable, can be quickly harvested and are immune privileged and non-tumorigenic [7,12,13,14,15]. hAECs have been shown to reduce injury and improve functional outcomes in adult stroke models. We have previously shown that a single bolus of hAECs administered either 2 or 24 h after asphyxia induced by 25 min of complete umbilical cord occlusion in preterm fetal sheep significantly improved neuronal survival in the hippocampus, striatum and thalamus, partially improved myelination and was profoundly anti-inflammatory, attenuating the increase in microglia in both white and grey matter [16]. Furthermore, we have also previously shown that delayed intranasal infusion of hAECS given on days one, 3 and 10 after 25 min of complete umbilical cord occlusion was associated with restored oligodendrocyte maturation and myelination as well as reduced inflammation as seen by a reduction in microglial and astrocyte numbers and attenuation of neuronal loss in the striatum after 21 days of recovery in preterm fetal sheep [17]. In near-term fetal sheep, hAECs given 0, 6 and 12 h after fetal LPS exposure [18], and to preterm sheep fetuses daily starting a day after first LPS exposure [19], significantly reduced microglial infiltration, pyknotic cells, apoptosis and oligodendrocyte loss. hAECs given to postnatal day 4 mice after prenatal LPS exposure and postnatal hyperoxia was also associated with reduced apoptosis and astrocytes and increased activated microglia [20]. However, to date, hAECs have not been examined after hypoxia–ischemia in the near-term fetal sheep. In fact, there are strikingly few studies of the neuroprotective effects of any type of stem cells in a term-equivalent model of moderate to severe hypoxic–ischemic brain injury.

In this study, we examined the hypothesis that hAECs given i.v. to near-term (0.85 gestational age) fetal sheep at 2 h after cerebral ischemia would confer both grey and white matter protection as evaluated at 7 days post-ischemia. The neural maturation of 0.85 ga fetal sheep is broadly equivalent to term brain maturation in human development, allowing us to investigate potential human neonatal treatment strategies, while the lamb is still in utero and therefore does not require intensive care level support [21,22].

## 2. Results

There was no significant difference in the sex distribution, number of singletons to twins and post-mortem body or brain weights between the sham control, ischemia-vehicle and ischemia-hAEC groups (Table 1, *p* > 0.05).

The baseline blood gases, acid-base and glucose-lactate values for all fetuses were in the normal range by our laboratory standards and did not significantly differ between groups (Table 2), other than a significantly higher pCO_2_ in the ischemia-hAEC group compared with sham control and ischemia-vehicle, which remained significantly elevated compared with ischemia-vehicle at all time-points except on day 5 and day 7 and compared with sham control at 2 h, 4 h and 6 h, day 1 and day 7 (*p* < 0.05). pH was significantly reduced in the ischemia-hAEC group at 2 h and 4 days compared with sham control and at 4 h, 6 h and 4 days compared with ischemia-vehicle (*p* < 0.05). Lactate was significantly elevated in the ischemia-vehicle group from 2 h to day one and in the ischemia-hAEC group from 2 h to day 2 compared with the sham control group (*p* < 0.05), with no significant differences between the ischemia-vehicle and ischemia-hAEC groups (*p* > 0.05). Glucose was significantly elevated in the ischemia-vehicle group from 2 h until day 1 in the ischemia-hAEC group at 2 h, 4 h, 6 h and day 2 compared with sham control (*p* < 0.05) with no significant differences between the ischemia-vehicle and ischemia-hAEC groups (*p* > 0.05).

### 2.1. Physiology

**Figure 1:**
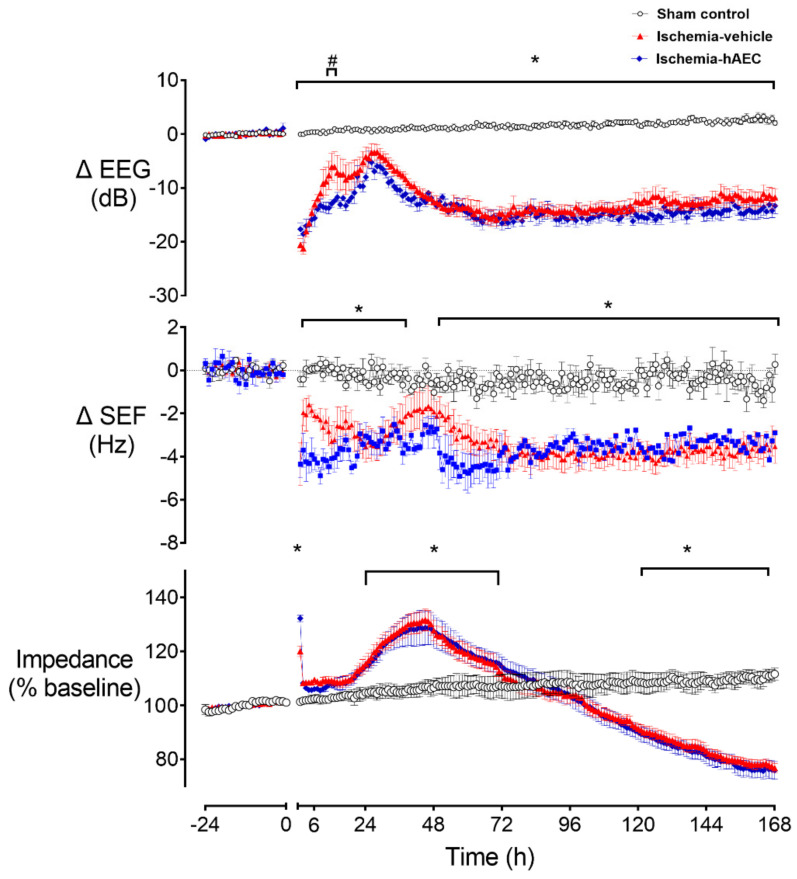
Changes in electroencephalogram (EEG) power, spectral edge frequency (SEF) and impedance before and after 30 min of global cerebral ischemia in near-term fetal sheep in the sham control (n = 8), ischemia-vehicle (n = 7) and ischemia-hAEC groups (n = 6). Data are 1 h averaged mean ± SEM * *p* < 0.05 vs. sham control, # *p* < 0.05 vs. ischemia-vehicle group.

Ischemia was associated with a significant reduction in electroencephalogram (EEG) power and spectral edge frequency (SEF) and a significant increase in cortical impedance compared with the sham control group (*p* < 0.05, Figure 1). EEG power remained significantly below sham control levels until the end of the recovery period and was transiently higher in the ischemia-vehicle group compared with the ischemia-hAEC group between 12–13 h (*p* < 0.05). SEF was significantly lower in the ischemia-vehicle and ischemia-hAEC groups from the end of occlusion until 30 h after occlusion and again from 48 h until the end of the experiment compared with the sham control group (*p* < 0.05). Impedance was significantly increased in both ischemia groups between 24 and 72 h and significantly reduced from 120 h until the end of the experiment in both ischemia groups compared with sham control (*p* < 0.05).

Seizures started earlier in the ischemia-hAEC group compared with the ischemia-vehicle group (7.8 ± 2.1 h vs. 17.4 ± 3.0 h, *p* < 0.05), but both groups had a similar total seizure burden: ischemia-hAEC group 5.7 ± 2.6 h vs. 5.0 ± 1.9 h in the ischemia-vehicle group, and these seizures occurred over a time period of 56.2 ± 8.6 h ischemia-hAEC group vs. 37.3 ± 9.8 h in the ischemia-vehicle group. No seizures were observed in the sham control group.

**Figure 2:**
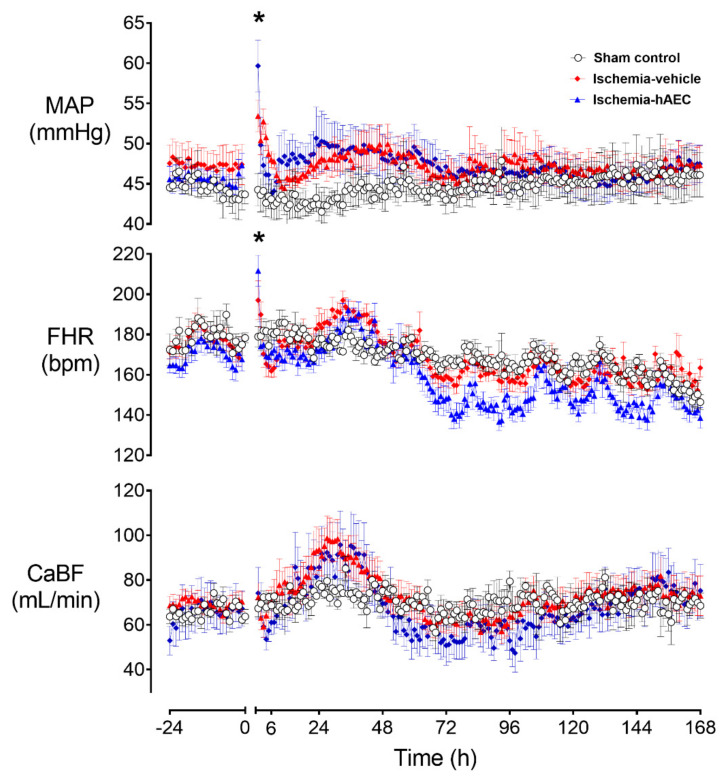
Changes in mean arterial pressure (MAP), fetal heart rate (FHR) and carotid artery blood flow (CaBF) before and after 30 min of global cerebral ischemia in near-term fetal sheep in the sham control (n = 8), ischemia-vehicle (n = 7) and ischemia-hAEC groups (n = 6). Data are 1 h averaged mean ± SEM * *p* < 0.05 vs. sham control.

Ischemia was associated with a significant increase in mean arterial pressure (MAP) and fetal heart rate (FHR) and decrease in carotid artery blood flow (CaBF), with both MAP and FHR remaining significantly elevated one hour after the end of ischemia before returning to sham control level for the remainder of the experiment (*p* < 0.05, Figure 2).

### 2.2. Histology

**Figure 3:**
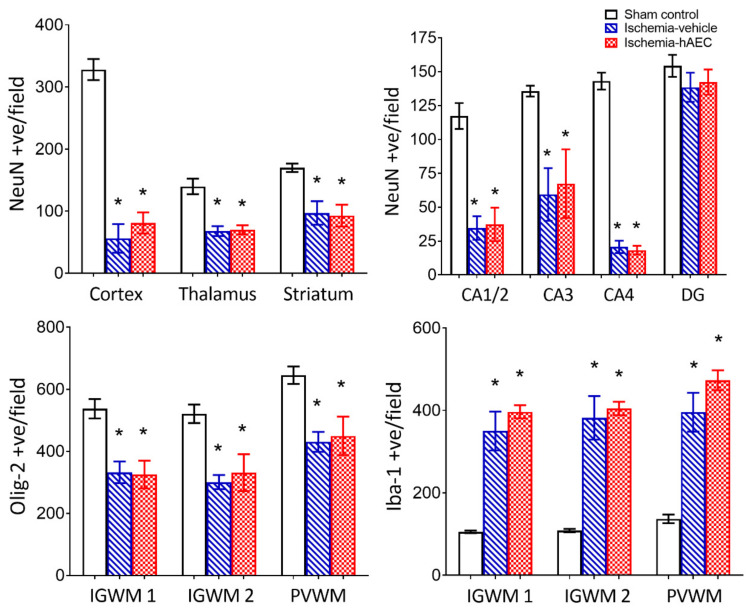
Neuronal number in the cortex, thalamus and striatum (**top left**) and CA1/2, CA3, CA4 and dentate gyrus of the hippocampus (**top right**), Olig2-positive total oligodendrocyte number (**bottom left**) and Iba1-positive microglial number (**bottom right**) in the sham control (n = 8), ischemia-vehicle (n = 7) and ischemia-hAEC groups (n = 6). Data are mean ± SEM * *p* < 0.05 vs. sham control.

**Figure 4:**
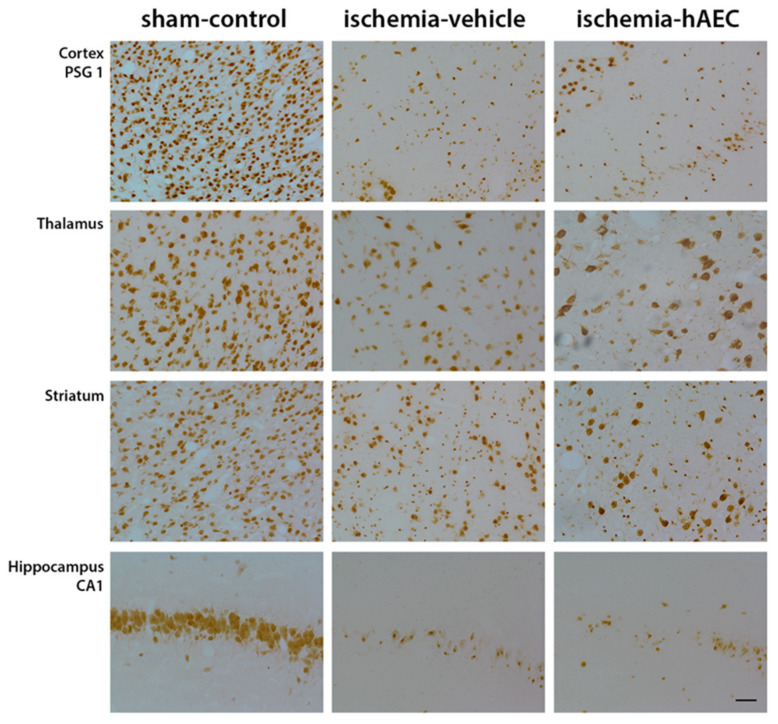
Photomicrographs showing representative NeuN-labelled images in the cortex, thalamus, striatum and CA1 of the hippocampus in the sham control, ischemia-vehicle and ischemia-hAEC groups. Scale bar is 50 µm.

**Figure 5:**
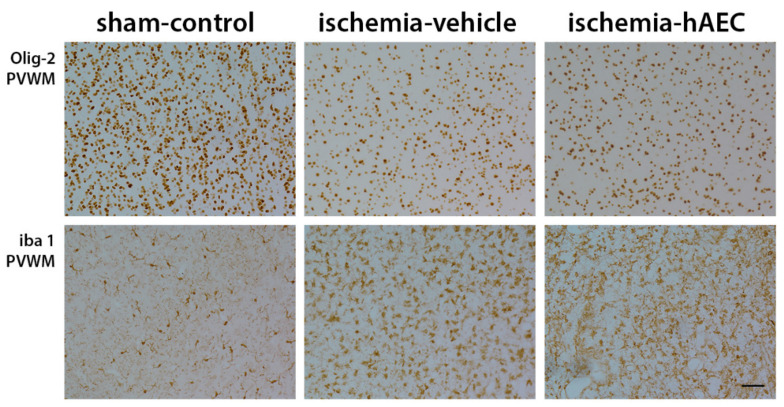
Photomicrographs showing representative Olig-2- and Iba1-labelled images in the periventricular white matter in the sham control, ischemia-vehicle and ischemia-hAEC groups. Scale bar is 50 µm.

Ischemia was associated with loss of neurons in the cortex, thalamus, striatum and CA1/2, CA3, CA4 and dentate gyrus of the hippocampus compared with sham control (*p* < 0.05), with no significant difference between the ischemia-vehicle and ischemia-hAEC groups (*p* > 0.05, Figure 3 and Figure 4). Ischemia was associated with loss of Olig-2-positive oligodendrocytes and an increase in Iba1-positive microglia in the intragyral white matter of the first and 2nd parasagittal gyrus and periventricular white matter compared with sham control (*p* < 0.05), with no significant difference between the ischemia-vehicle and ischemia-hAEC groups (*p* > 0.05, Figure 3 and Figure 5).

## 3. Discussion

This study shows that a single intravenous bolus infusion of 52.7 ± 6.5 × 10^6^ human amnion epithelial cells administered two hours after the end of 30 min of global cerebral ischemia was not associated with any beneficial effect on the electrophysiological or cardiovascular recovery or the development of brain damage, as seen by loss of neurons and oligodendrocytes and increase in microglial number on day 7 in near-term fetal sheep.

The lack of any neuroprotective or anti-inflammatory effects in this study is surprising. We have previously shown that a single intracerebroventricular bolus of 1 × 10^6^ hAECs administered either 2 or 24 h after asphyxia induced by complete umbilical cord occlusion in preterm fetal sheep significantly improved neuronal survival in the hippocampus, partially improved myelination and was profoundly anti-inflammatory, attenuating the increase in microglia in both white and grey matter [16]. However, neither protocol improved recovery of EEG power or oligodendrocyte survival and only treatment started at 24 h, but not 2 h, improved neuronal survival in the striatum and thalamus., Furthermore, we have also previously shown that delayed intranasal infusion of 40.7 × 10^6^ hAECS given on days one, 3 and 10 after 25 min of complete umbilical cord occlusion was associated with restored oligodendrocyte maturation and myelination as well as reduced inflammation as seen by a reduction in microglial and astrocyte numbers and attenuation of neuronal loss in the striatum but no improvement in the recovery of EEG power over 21 days of recovery in preterm fetal sheep [17]. Consistent between all 3 studies, hAEC administration was not associated with any improvement in the recovery of EEG power or in number of total surviving oligodendrocytes. Intriguingly, both hAEC administration protocols, whether started at 2 or 24 h after the insult, were profoundly anti-inflammatory in the preterm fetal sheep but not in the term equivalent fetal sheep. The lack of an anti-inflammatory effect seen in the current study is consistent with a study in 0.8 gestation lambs, in which umbilical cord blood cell administration given one hour after the insult had no effect on immune cell infiltration or lung injury in lambs who received injurious ventilation after birth [23]. The authors proposed that the lack of beneficial effects seen in this study may have been attributed to their introducing stem cells during the peak inflammatory period and indeed it has been shown that mesenchymal stromal cells can exacerbate the inflammatory response if introduced into an established pro-inflammatory environment [23,24]. The time course of the inflammatory response after HI in the near-term fetal sheep is not well characterized but it is possible that administration of hAECs either earlier or later in the evolution of injury may have had benefit.

There are several differences between the current study and the 2 described above performed in preterm fetal sheep that may underlie the discrepancy in these results. Critically, in the term-equivalent brain, 30 min of global cerebral ischemia is associated with severe neuronal loss in the cortex as well as subcortical regions, while in the preterm brain, no significant cortical neuronal loss develops and subcortical neuronal loss is much less extensive. Therefore, neuronal injury in the term brain may be too severe to be salvaged by treatment with hAECS given that treatment started at 2 h in the preterm brain was only associated with neuronal protection in the hippocampus.

Next, it is not clear how the route of administration would affect the number of hAECs that reached the brain and their efficacy. The current study is one of the first studies of perinatal brain injury to give hAECs intravenously rather than intracerebroventricularly or intranasally [16,17]. Interestingly, in a study in newborn piglets after hypoxia ischemia, intranasal administration of mesenchymal stromal cells was associated with modest augmentation of hypothermic neuroprotection, including better recovery of amplitude integrated EEG and increased oligodendrocyte number in the hippocampus, internal capsule and periventricular white matter compared with when cells were administered intravenously [25]. It has previously been reported that after hypoxia ischemia in adult mice, neural stem cells transduced with the firefly luciferase reporter gene for bioluminescence imaging, 94% of the bioluminescence signal was detected in the lungs after intravenous injection cell suggesting that only a very small proportion of cells successfully pass through the pulmonary circulation [26]. However, after intra-arterial administration, 69% of the bioluminescence signal arose from the brain, suggesting this may be a more effective route of administration. In contrast, a number of preclinical/studies have shown beneficial effects of intravenous administration of stem cells [27]. Intriguingly, these studies also suggest that intra-arterial administration of stem cells is more effective than intravenous, suggesting that intra-arterial administration of hAECs should be further investigated in perinatal brain injury models in the future. In P10 neonatal rat pups, intracerebroventricular infusion of human umbilical cord mesenchymal stem cells, administered one hour after hypoxia–ischemia induced via the Rice–Vannucci model, significantly ameliorated brain damage, including reducing infarct volume and apoptotic cell death at 48 h, and improved cognitive and motor function at 28 days, compared with untreated pups [28]. However, intracerebroventricular administration would be difficult to translate to the clinic, unlike intravenous and potentially intranasal routes of administration. Interestingly, a small first in human study in 10 neonates, presenting with perinatal arterial ischemic stroke, demonstrated that intranasal bone marrow derived mesenchymal stromal cell administration is feasible in neonates, with no evidence of serious adverse effects [29].

It is likely that the number and timing of treatments modulates the extent of neuroprotective benefit. It is plausible that repeated injections of hAECs may be more likely to show an effect than a single injection, given that we saw restoration of oligodendrocyte maturation after 3 delayed intranasal treatments with hAECs but not after a single intracerebroventricular treatment in preterm fetal sheep [16,17]. Similarly, 3 doses of umbilical cord blood cells administered via either intraperitoneal or intranasal injection on day 11, 13 and 20, reduced loss of tissue and attenuated neuropathology, including apoptosis, and neuronal loss and microglial up-regulation, where as a single dose administered on postnatal day 11 had no effect after hypoxia ischemia induced using the Rice–Vannucci model at P10 [30]. Therefore, it is possible that repeated treatments with hAECs may have may been more likely to show an effect than the single treatment given in the current study.

Limitations of this study include the fact that only a single dose and single route of administration were tested. Future studies should investigate the effectiveness of multiple doses of hAECs, different timings of administration, other routes of administration, particularly intranasal administration as well as determine whether administration of hAECs may have an additive effect when given with or after therapeutic hypothermia.

In conclusion, a single intravenous infusion of hAECs was not associated with any protective effects on neurophysiological or histological parameters in the near-term fetal sheep after global cerebral ischemia.

## 4. Methods and Materials

Women gave written, informed consent for the collection of their placentae, and all procedures conformed to the standards set by the *Declaration of Helsinki*. Human amnion epithelial stem cells were collected from the placentae of women with uncomplicated pregnancies undergoing elective caesarean section at term. Tissue collection was performed with approval from the Monash Health Human Research Ethics Committee, Monash, Melbourne, Australia.

All animal procedures were approved by the Animal Ethics Committee of the University of Auckland. Time-mated Romney/Suffolk fetal sheep were instrumented using sterile technique at 124–126 days ga (term is 145 days) [31,32]. Ewes were anesthetized by intravenous injection of propofol (5 mg/kg, AstraZeneca Limited, Auckland, New Zealand), followed by 2–3% isoflurane in oxygen. Ewes received a constant infusion isotonic saline drip during surgery and 1 mL/10 kg oxytetracycline (200 mg/mL, Phoenix Pharm Distributors Ltd., Auckland, New Zealand) i.m. for prophylaxis just prior to surgery.

The fetus was partially exposed after maternal midline abdominal and uterine incision, and the brachial arteries and brachial veins were catheterized with polyvinyl catheters to allow for mean arterial blood pressure monitoring and preductal blood sampling and infusions. An amniotic catheter was secured to the fetal shoulder. Electrocardiogram (ECG) electrodes (Cooner Wire Co., Chatsworth, CA, USA) were sewn across the fetal chest to record fetal heart rate. The carotid anastomoses were ligated, and occluder cuffs were placed around both common carotid arteries as previously described [33]. An ultrasonic flow probe (3S type, Transonic Systems Inc., Ithaca, NY, USA.) was placed around the right carotid artery as to provide an index of cephalic blood flow [34,35]. Two pairs of electrodes were implanted biparietally to record brain electroencephalographic (EEG) activity and seizures, a pair of impedance electrodes were placed 5 mm lateral of the EEG to measure cortical impedance (Cooner Wire Co., Chatsworth, CA, USA). The uterus was then closed and antibiotics (80 mg Gentamicin, Pharmacia and Upjohn, Rydalmere, Australia) were administered into the amniotic sac. The maternal laparotomy skin incision was infiltrated with a local analgesic, 10 mL 0.5% bupivacaine plus adrenaline for analgesia (AstraZeneca Ltd., Auckland, New Zealand). All fetal catheters and leads were exteriorized through the maternal flank. The maternal long saphenous vein was catheterized for post-operative care and euthanasia.

### 4.1. Post-Operative Care

Sheep were housed together in separate metabolic cages with access to food and water ad libitum. They were kept in a temperature-controlled room (16 ± 1 °C, humidity 50 ± 10%), in a 12 h light/dark cycle. Antibiotics were given daily for four days i.v. to the ewe (600 mg benzylpencillin, Novartis Ltd., Auckland, New Zealand, and 80 mg Gentamicin, Pharmacia and Upjohn). Fetal catheters were maintained patent by continuous infusion of heparinized saline (20 U/mL at 0.15 mL/h) and the maternal catheter was maintained patent by daily flushing.

### 4.2. Experimental Protocol

On day 129 ± 1 of gestation, fetuses were randomly assigned to one of the following groups: sham-sham group (n = 8), ischemia-vehicle group (n = 7), ischemia-hAEC group (n = 6). Fetal ischemia was induced by completely occluding both carotids for 30 min. Successful occlusion was determined by rapid onset isoelectric EEG, and a rise in cortical impedance at proximately 5 min. All occlusions were started in the morning between 9 and 9.30 a.m.

Fetal arterial blood samples were collected at 1 h before, and 2, 4 and 6 h after the end of occlusion. Then, samples were taken daily thereafter between 8 and 9 am. Fetal pre-ductal arterial pH, PCO_2_, PO_2_ (Ciba-Corning Diagnostics 845 blood gas analyzer and co-oximeter, East Walpole, MA, USA), and glucose and lactate (YSI model 2300, Yellow Springs, OH, USA) measurements were made. The intravenous infusion of 52.7 ± 6.5 × 10^6^ cells suspended in 2 mL sterile PBS was administered 2 h after occlusion termination and the catheter flushed with a further 3 mL. Ischemia-vehicle fetuses received the same volume of saline at the same time.

### 4.3. Cell Preparation

The cells were prepared as previously published in a methods paper by Murphy et al. [36]. The hAECs were provided by The Ritchie Centre, Melbourne and delivered frozen. Before administration, cells were defrosted and placed in culture overnight. Cells were then removed from culture washed and assessed for number and viability before 52.7 ± 6.5 × 10^6^ cells were suspended in 2 mL sterile PBS for administration [37]. The dose was based on extrapolation from previous studies in fetal sheep [16,18,19].

### 4.4. Immunohistochemistry

The fetal brains were perfusion fixed with 10% phosphate-buffered formalin at day 7 and embedded in paraffin. Slices (10 μm thick) were cut using a microtome (Leica Jung RM2035, Wetzlar, Germany) and immunohistochemistry was performed as previously described [38]. Slides were dewaxed and rehydrated. Citrate antigen retrieval was performed for all antigens, followed by endogenous peroxidase quenching. Blocking was performed with 3% normal horse serum in PBS for Iba-1 or 3% normal goat serum in PBS for all other stains for 1 h at room temperature. Sections were labelled with monoclonal primary antibodies: 1:200 concentration rabbit anti-ionized calcium binding adaptor molecule 1 (Iba-1, a marker for activated microglia, Abcam, Cambridge, MA, USA). 1:400 rabbit anti-oligodendrocyte transcription factor 2 (Olig2, a marker for all stages of the oligodendrocyte lineage, Merck-Millipore Corporation, Billerica, MA, USA), 1:200 rabbit anti-neuronal nuclear antigen (NeuN, a marker of mature neurons, Merck-Millipore, Burlington, MA, USA).

Sections labelled with these primary antibodies were incubated overnight at 4 °C, then washed and incubated with secondary biotinylated monoclonal anti-rabbit 1:200 IgG antibodies (Vector Laboratories, Burlingame, CA, USA) for 3 h at room temperature. Slides were incubated in ExtrAvidin 1:200 (Sigma Aldrich, St. Louis, Mo, United States) for 2 h at room temperature and reacted with DAB (Sigma Aldrich). Neurons were assessed in three areas of the cortex, five areas in the striatum and thalamus and in the CA1, CA2/3, CA4 and DG of the hippocampus. Oligodendrocytes and microglia were assessed in the intragyral and periventricular white matter. Light microscopy was used for image acquisition at ×40 or ×20 magnification on a Nikon 80i microscope with a motorized stage. Cell number was averaged between left and right hemispheres of 2 slides for statistical analysis and graphing.

### 4.5. Data Analysis

Fetal parameters were recorded continuously from 24 h before, until the end of the experiment. Data were processed using Labview (National Instruments, Austin, TX, USA) and raw and one minute averaged data were stored to computer disk, these were averaged into hourly Means ± SEM for statistical analysis and graphing. Seizures were quantified as total time spent having seizures, start time of seizures and total period over which seizures occurred, as previously described [39].

### 4.6. Statistics

Data were compared between groups using repeated measures or one-way analysis of variance (ANOVA) followed by the least significant difference *post hoc* tests when a significant effect of group was found (SPSS v22, SPSS Inc., Chicago, IL, USA). Statistical significance was accepted at *p* < 0.05. Data are mean ± standard error of the mean (SEM).

## Figures and Tables

**Table 1:** Sex distribution, number of singletons and twins per group and post-mortem body and brain weight for the sham control (n = 8), ischemia-vehicle (n = 7) and ischemia-hAEC (n = 6) groups. F = female, M = male, U = unknown.

Group	Sex	Singleton/Twin	Body Weight (g)	Brain Weight (g)
**Sham Control**	5F3M	8S	4588.3.3 ± 359	40.1± 1.4
**Ischemia-vehicle**	3M3F1U	6S1T	4697.1.3 ± 240	39 ± 1.2
**Ischemia-hAEC**	5F1M	6S	4112.3 ± 238	38 ± 1.4

**Table 2:** Blood gas, glucose and lactate concentration before, 2 h, 4 h and 6 h after ischemia, followed by daily until day 7 in near term fetal sheep in the sham control (n = 8), ischemia-vehicle (n = 7) and ischemia-hAEC groups (n = 6). * *p* < 0.05 versus sham control, ^#^ *p* < 0.05 versus ischemia-vehicle.

Group		Baseline	2 h	4 h	6 h	1 d	2 d	3 d	4 d	5 d	6 d	7 d
**Sham-control**	**pH**	7.38 ± 0.01	7.38 ± 0.01	7.38 ± 0.01	7.39 ± 0.01	7.38 ± 0.01	7.37 ± 0.01	7.37 ± 0.01	7.36 ± 0.01	7.35 ± 0.01	7.34 ± 0.01	7.35 ± 0.01
**Ischemia-vehicle**		7.4 ± 0.01	7.39 ± 0.01	7.41 ± 0.01	7.40 ± 0.01	7.38 ± 0.02	7.39 ± 0.01	7.37 ± 0.01	7.37 ± 0.01	7.37 ± 0.01	7.37 ± 0.01	7.37 ± 0.01
**Ischemia-hAEC**		7.34 ± 0.03	7.32 ± 0.03 *	7.33 ± 0.03 ^#^	7.35 ± 0.02 ^#^	7.35 ± 0.02	7.35 ± 0.01	7.34 ± 0.01	7.31 ± 0.01 *^,#^	7.33 ± 0.01	7.33 ± 0.02	7.35 ± 0.01
**Sham-control**	**pCO_2_**	48.8 ± 4.3	47.4 ± 2.8	48.8 ± 1.5	47.3 ± 0.1	47.2 ± 1.1	48.3 ± 1.3	46.4 ± 1.2	48.7 ± 0.7	50.3 ± 0.5	49.5 ± 1.8	46.8 ± 0.1
**Ischemia-vehicle**		44.6 ± 1.4	42.6 ± 1.6	43.4 ± 1.4	44.9 ± 1.9	44.4 ± 2.7	44.1 ± 1.3	44.5 ± 1.9	45.5 ± 1.2	46.7 ± 1.9	46.7 ± 1.6	49.4 ± 1.9 *
**Ischemia-hAEC**		55.3 ± 1.3 *^,#^	52.3 ± 1.3 *^,#^	53.1 ± 1.2 *^,#^	54.0 ± 1.0 *^,#^	54.2 ± 0.8 *^,#^	52.8 ± 1.3 ^#^	53 ± 1.3 ^#^	54.2 ± 1.2 ^#^	51.8 ± 1.4	55.5 ± 1.2 ^#^	53.9 ± 1.0 *
**Sham-control**	**pO_2_**	21.4 ± 0.3	21.6 ± 1.2	21.2 ± 1.1	20.9 ± 1.2	20.1 ± 1.3	22.6 ± 3.1	22.2 ± 2.9	22.9 ± 2.2	24.3 ± 3.6	26 ± 2.7	23.7 ± 2.2
**Ischemia-vehicle**		26.0 ± 1.1	25.8 ± 1.2	25.9 ± 1.3	25.4 ±1.9	25.0 ± 2.0	24.6 ±1.5	26.7 ± 1.6	25.5 ± 1.9	26.0 ± 1.9	24.7 ± 1.4	22.9 ± 1.1
**Ischemia-hAEC**		22.9 ± 1.3	20.6 ± 1.2	20.1 ± 0.6	20.6 ± 1.0	19.1 ± 1.2	19.5 ± 1.4	26.9 ± 1.0	26.6 ± 0.9	24.8 ± 1.2	24.3 ± 1.2	24.2 ± 0.8
**Sham-control**	**Lactate**	0.9 ± 0.2	1.0 ± 0.2	1.2 ± 0.0	1.2 ± 0.1	1.0 ± 0.1	1.0 ± 0.2	0.9 ±0.4	1.0 ± 0.2	0.8 ± 0.2	0.8 ± 0.3	0.8 ± 0.4
**Ischemia-vehicle**		1.1 ± 0.1	2.4 ± 0.5 *	2.3 ± 0.5 *	2.1 ± 0.4 *	4.7 ± 1.1 *	0.9 ± 0.1	1.0 ± 0.1	1.1 ± 0.1	1.1 ± 0.1	1.1 ± 0.1	1.2 ± 0.1
**Ischemia-hAEC**		1.2 ± 0.1	3.8 ± 0.7 *	3.9 ± 0.8 *	3.5 ± 0.7 *	2.5 ± 0.7 *	2.6 ± 0.7 *	0.8 ± 0.1	0.8 ± 0.1	0.9 ± 0.1	0.9 ± 0.1	0.9 ± 0.1
**Sham-control**	**Glucose**	0.6 ± 0.3	0.6 ± 0.2	0.8 ± 0.2	0.8 ± 0.2	0.8 ± 0.2	0.7 ± 0.1	0.6 ± 0.3	0.6 ± 0.1	0.6 ± 0.2	0.6 ± 0.1	0.5 ± 0.2
**Ischemia-vehicle**		1.0 ± 0.1	1.3 ± 0.1 *	1.3 ± 0.1 *	1.3 ± 0.1 *	1.5 ± 0.1 *	0.9 ± 0.1	0.9 ± 0.1	0.9 ± 0.1	1.0 ± 0.1	1.0 ± 0.1	1.0 ± 0.1
**Ischemia-hAEC**		1.0 ± 0.1	1.6 ± 0.1 *	1.4 ± 0.1 *	1.4 ± 0.1 *	1.1 ± 0.2	1.3 ± 0.3 *	0.8 ± 0.1	0.8 ± 0.1	0.9 ± 0.1	0.8 ± 0.1	0.9 ± 0.1

## Data Availability

Data are available from the corresponding author on reasonable request.
